# Suboptimal Porcine Endogenous Retrovirus Infection in Non-Human Primate Cells: Implication for Preclinical Xenotransplantation

**DOI:** 10.1371/journal.pone.0013203

**Published:** 2010-10-06

**Authors:** Giada Mattiuzzo, Yasuhiro Takeuchi

**Affiliations:** Division of Infection and Immunity, Wohl Virion Centre, University College London, London, United Kingdom; Veterinary Laboratories Agency, United Kingdom

## Abstract

**Background:**

Porcine endogenous retrovirus (PERV) poses a potential risk of zoonotic infection in xenotransplantation. Preclinical transplantation trials using non-human primates (NHP) as recipients of porcine xenografts present the opportunity to assess the zoonosis risk *in vivo.* However, PERV poorly infects NHP cells for unclear reasons and therefore NHP may represent a suboptimal animal model to assess the risk of PERV zoonoses. We investigated the mechanism responsible for the low efficiency of PERV-A infection in NHP cells.

**Principal Findings:**

Two steps, cell entry and exit, were inefficient for the replication of high-titer, human-tropic A/C recombinant PERV. A restriction factor, tetherin, is likely to be responsible for the block to matured virion release, supported by the correlation between the levels of inhibition and tetherin expression. In rhesus macaque, cynomolgus macaque and baboon the main receptor for PERV entry, PERV-A receptor 1 (PAR-1), was found to be genetically deficient: PAR-1 genes in these species encode serine at amino acid 109 in place of the leucine in human PAR-1. This genetic defect inevitably impacts *in vivo* sensitivity to PERV infection of these species. In contrast, African green monkey (AGM) PAR-1 is functional, but PERV infection is still poor. Although the mechanism is unclear, tunicamycin treatment, which removes N-glycosylated sugar chains, increases PERV infection, suggesting a possible role for the glycosylation of the receptors.

**Conclusions:**

Since cynomolgus macaque and baboon, species often used in pig-to-NHP xenotransplantation experiments, have a defective PAR-1, they hardly represent an ideal animal model to assess the risk of PERV transmission in xenotransplantation. Alternatively, NHP species, like AGM, whose both PARs are functional may represent a better model than baboon and cynomolgus macaque for PERV zoonosis *in vivo* studies.

## Introduction

The control of potential risk of zoonosis is a prerequisite for the development of clinical xenotransplantation. Potential transmission to xenotransplantation recipients and further spread to the general public of porcine endogenous retroviruses (PERV) has been a major concern in the xenotransplantation field [1], [2], [3]. PERVs are present in the pig genome in the form of provirus DNA. These proviruses are descendants of viral DNA integrated in the germ line chromosome by ancient exogenous retroviral infections and most of them have become defective through evolution [4], [5], [6], [7]. However, certain intact PERVs can infect human cells *in vitro*, posing a potential risk of zoonosis in pig-to-human xenotransplantation [8], [9], [10]. Pig genomes have 50–100 PERV copies, and their integration pattern is highly polymorphic. This makes it very difficult, if not impossible, to remove PERV sequences from transplantation source pigs [8], [11], [12], [13], [14], [15], [16]. Although PERV infection has not been detected in retrospective analysis of patients treated with porcine cells and tissues [17], [18], [19], [20], [21], [22], this risk cannot be excluded in xenotransplantation. Longer exposure to PERV of less immunosuppressed patients in successful xenotransplantation would increase the chance of PERV transmission. Therefore, development of transplantation source animals with reduced PERV activity, development and refinement of clinical tests for PERV transmission, and accurate preclinical risk assessment, are much needed.

Three subgroups of PERVs with distinctive *env* genes have been identified in pig genomes [5], [6]. PERV-A and PERV-B can infect several species including human cells, while PERV-C tropism is limited to pig cells [9], [23]. However, it has been shown that recombination between PERV-A and PERV-C occurs frequently, producing a high titer, human tropic PERVA/C [23], [24]. These recombinant PERVs use the same receptor for cell entry as PERV-A, and they are almost exclusively the form of isolates derived by co-cultivation of porcine primary cells and human cells [10], [24], [25]. This form is therefore considered to be most problematic [2], [3], [26].

Ongoing preclinical transplantation trials using non-human primates (NHP) as recipients of porcine cells, tissues, and organs present the opportunity to assess the zoonosis risk *in vivo* [27], [28], [29], [30], [31], [32]. NHP offer the opportunity to evaluate the risk of PERV transmission after a long exposure to the pig xenograft, a variety of tissue can be analysed and the immunosuppression required can be simulated. However, the use of NHP to assess the risk of PERV transmission has been debated. Initial studies showed that NHP cell lines were not permissive for PERV infection [8], [9], [23], [33]. Other reports, which used sensitive PCR or RT-PCR to detect PERV sequences, suggested that NHP cells are susceptible [34], [35], [36]. By using a high titer PERV derived from NIH miniswine animals, it was possible to show that PERV could infect rhesus macaque and African green monkey (AGM) cell lines. In the infected NHP cells, PERV provirus and transcripts were detected but no reverse transcriptase activity was found in the supernatant of these cells, suggesting that PERV infection of NHP cells was not productive [37]. However, the mechanism responsible for the poor infectivity and the lack of PERV replication in NHP cells remains unclear. Here, the reasons for the low susceptibility of NHP cells to PERV infection have been investigated. The implication of our results on the suitability of NHP models, and the choice of the NHP species for the study of PERV transmission, is discussed.

## Results

### The low PERV permissivity is mainly caused by reduced entry in NHP cells

**Figure 1:**
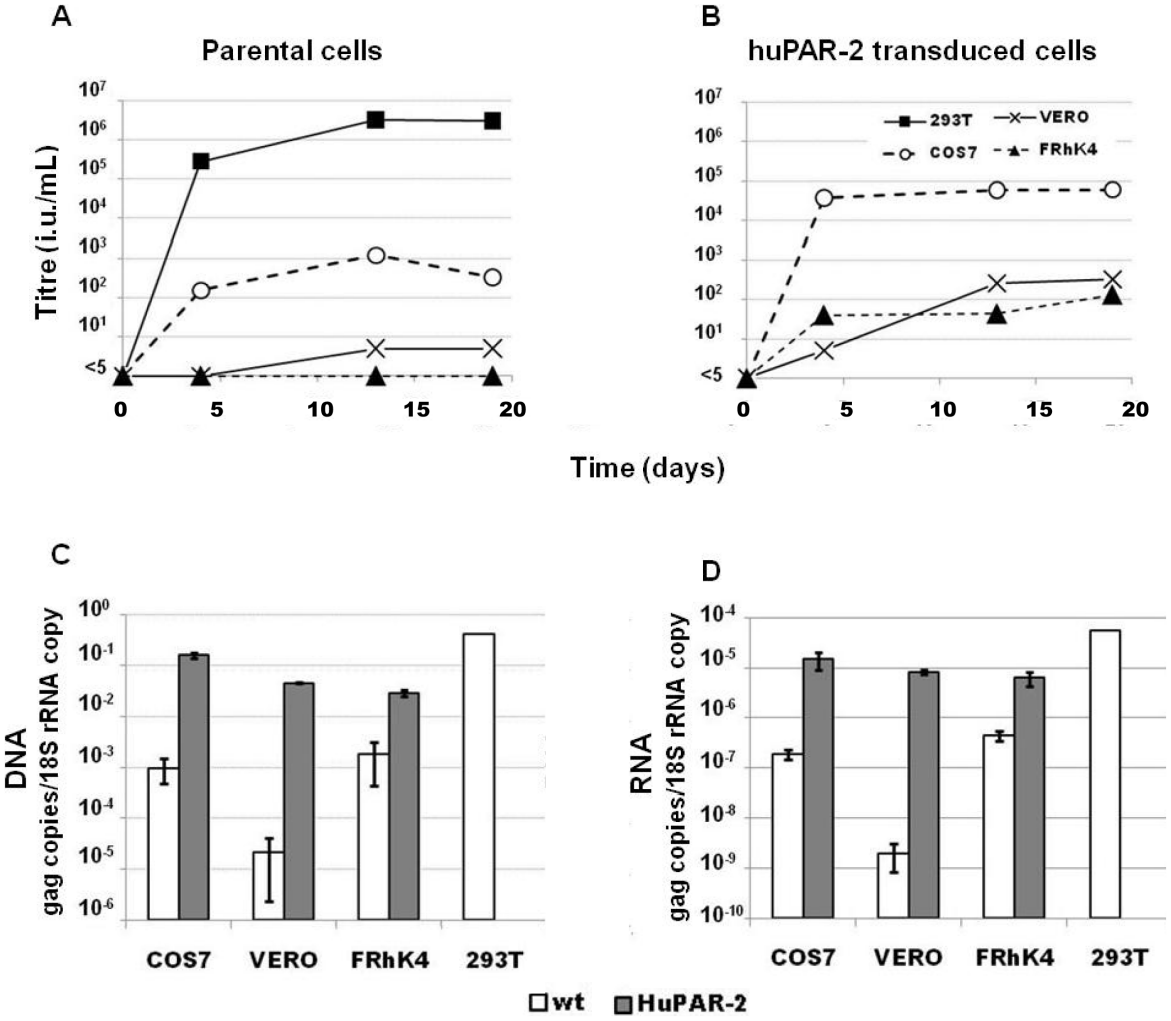
PERV-A infection analysis in NHP cell lines. HuPAR-2 gene was delivered into rhesus macaque FRhK4 cells, AGM COS7 and VERO cells using a VSV-G pseudotyped MLV-based vector. HuPAR-2 transduced or wild type NHP cells and human 293T cells were infected with PERV-A14/220 at MOI 20 (titer calculated on 293T cells). **A and B**) At the different time points indicated, 1×10^6^ PERV-infected cells FRhK4 (black triangle), COS7 (white circle), VERO (cross) and 293T (black square) cells were seeded. The day after, supernatant was collected and 5-fold dilutions used to infect 293T cells. 72 hrs later, cells were immunostained with an anti-PERV CA antibody. Data represent the average of two independent experiments (± standard error of the mean, SEM). **C**) One week post-infection, genomic DNA was extracted and copy numbers of PERV *gag* and 18S rRNA gene were measured by quantitative PCR. **D**) Two weeks post-infection, total RNA was extracted and RNA copy numbers of PERV *gag* and 18S ribosomal RNA were measured by quantitative RT-PCR. PERV *gag* copy number was calculated from standard curves and normalised per 18S rRNA copy. Each sample was run in duplicate. Histograms represent the average of two independent experiments (± SEM).

To determine whether PERV can productively infect NHP cells, progeny virus production was measured in a time course following PERV infection of African green monkey (AGM) COS7 and VERO cells, and rhesus macaque FRhK4 cells. The same cell lines, but also expressing huPAR-2 via a MLV-based retroviral vector, were also tested. Cells were infected with the replication-competent PERV-A14/220, a PERV-A/C recombinant, used as a representative of isolates derived from primary porcine cells [24], [38]. Cells were seeded in equal number at different time points after PERV infection, and the following day their supernatant was collected and the production of infectious PERV was determined by infection of human 293T cells [39]. PERV titers, obtained from NHP cells with or without huPAR-2, were lower than that by control human 293T cells. Infectious viruses were barely present in the supernatant of wild-type FRhK4 and VERO cells for up to three weeks post infection (Figure 1A). PERV titer from wild type COS7 and huPAR-2-transduced NHP cells was, however, detected, increasing in the first two weeks and stabilising at week three to various levels (Figure 1A–B). HuPAR-2 expression resulted in substantially higher PERV titer; in case of VERO cells to about 100 fold.

To define whether PERV replication is blocked in NHP cells at early or late stages of the virus life cycle, PERV integration and transcription in NHP cells were measured by quantitative PCR. PERV DNA was measured one week post infection (Figure 1C), while expression of PERV RNA was evaluated two weeks post infection (Figure 1D). The amount of PERV *gag* DNA copies in the parental cells were more than 100 times lower than that in human 293T cells, indicating that there is a major block in the early virus entry stage (Figure 1C, open bars). This block was alleviated by expression of huPAR-2, as PERV DNA was 10 to 1000 times higher in huPAR-2-transduced NHP (NHP/huPAR-2) cells than in parental cells (Figure 1C). PERV RNA expression levels, measured as *gag* RNA copies, corresponded to those of proviral DNA (compare Figure 1C and 1D). Overall, upon expression of a functional receptor, PERV-A can successfully enter the NHP cells and integrate in the host genome (Figure 1C), viral genes are transcribed (Figure 1D) and infectious particles produced (Figure 1B).

**Figure 2:**
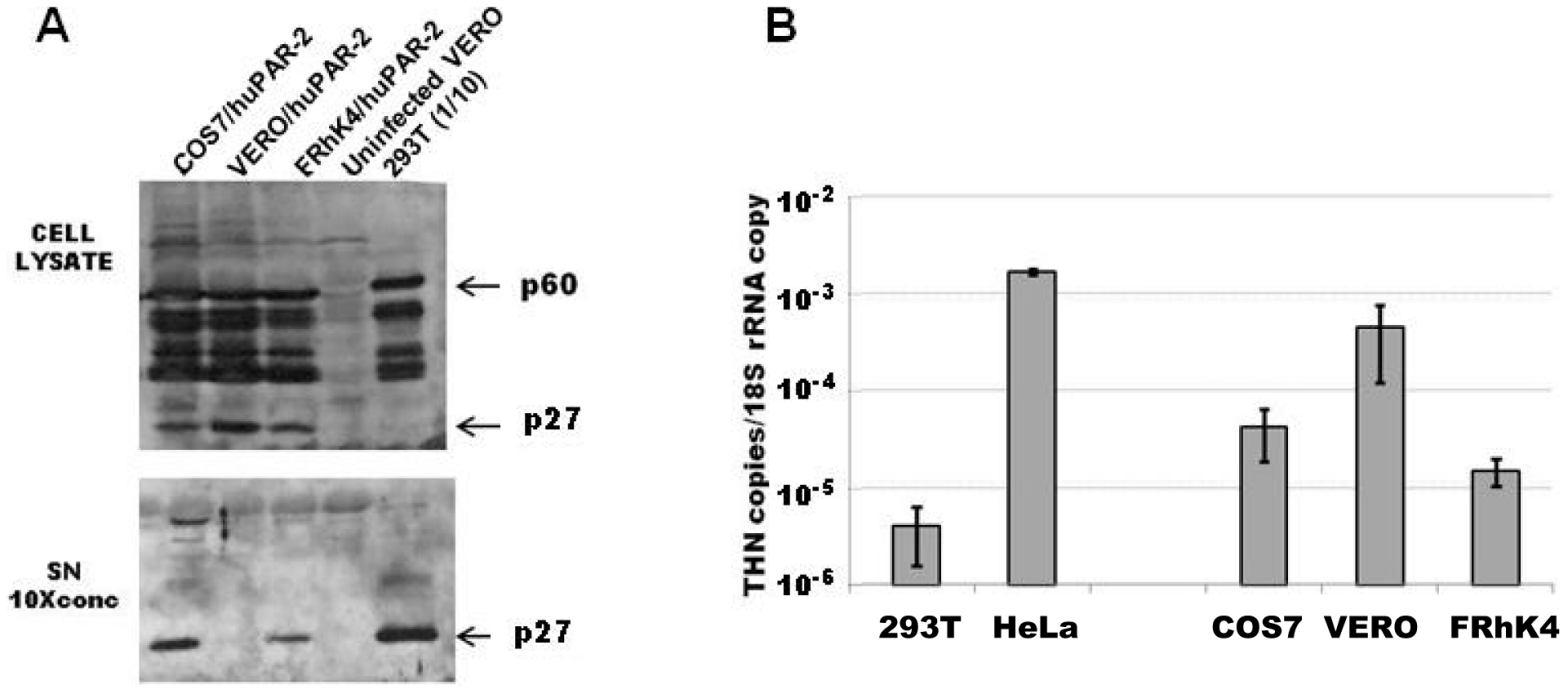
Inhibition of PERV release from NHP cells and expression of tetherin mRNA in NHP cell lines. **A**) HuPAR-2-transduced NHP cells or wild type human 293T cells were infected with PERVA14/220. Two weeks later, 1 mL of supernatant was harvested and concentrated 10 times. The same 1×10^6^ cells were lysed in radioimmunoprecipitation assay (RIPA) buffer. For NHP cells, one fourth of these cell lysate and supernatant samples were separated by 10% SDS-PAGE and immunoblotted using an anti-PERV CA antibody. Since PERV titer from 293T cells was more than 50 times higher than that from NHP cells, one fortieth of the 293T samples were loaded in the same gel, ten times less than that from NHP cells. The differently processed capsid forms were detected in the cell lysate (upper panel), while in the supernatant (SN) p27 was the major form (bottom panel). **B**) Tetherin RNA level in RNA from NHP FRhK-4, COS7 and VERO cells and human 293T and HeLa cells lines was quantified by quantitative RT-PCR. Samples were run in triplicate. The amount of copies for each gene was extrapolated from analysis of the standard curves. Histograms represent the mean of human (left bars) and NHP (right bars) tetherin copy number normalised to one 18S rRNA copy obtained by two independent experiments (± SEM).

However, the infectious titers produced by NHP/huPAR-2 cells, especially VERO/huPAR-2 and FRhK/huPAR-2, were still much lower than by human 293T cells (Figure 1A and B). This indicated that the amount of viral transcripts achieved in NHP/huPAR-2 cells did not correlate well with the infectious titer, and suggested that there may be another block to PERV replication in a late stage of the life cycle. Therefore, we examined PERV protein production in the NHP cell lysate and supernatant by western blot using an anti-PERV capsid antibody. Whilst similar amounts of Gag precursors were present, the amount of processed capsid (p27) in the cell lysate in NHP/huPAR-2 cells was much higher than in 293T cells (Figure 2A, cell lysate). In contrast, the amount of PERV p27 in the supernatant was lower in the NHP cells than in human 293T cells (Figure 2A, SN). These results suggest an inefficient release of ‘matured’ PERV from NHP cells. Tetherin (also known as BST-2, CD317 or HM1.24) is a restriction factor inhibiting the release of enveloped viruses from the producer cells by tethering the virions on the cell surface. The mRNA level of tetherin in NHP cell lines was quantified by real-time RT-PCR and compared to that in the human cell lines, 293T and HeLa, which express significantly different levels of human tetherin. While retroviral particles are successfully released from 293T cells, they are withheld on the cell surface of HeLa cells by a tetherin-mediated mechanism [40]. NHP cells express 10–100 times more tetherin mRNA than 293T cells (Figure 2B). In particular, VERO cells have only 5 times less mRNA than HeLa cells and they showed the highest amount of p27 in the cell lysate among NHP cells, whilst it was undetectable in the supernatant (Figure 2A). These results suggest that, in addition to the block at virus entry, a higher level of tetherin expression compared to that in 293T cells contributes to inefficient PERV replication in the NHP cells.

### NHP cells express a level of PERV-A receptors similar to human cells

PERV-A14/220 contains the receptor binding domain derived from PERV-A and therefore uses PERV-A receptors for cell entry [38], [41]. To investigate the reason behind the poor efficiency of PERV-A14/220 entry in NHP cells, we cloned the PERV-A receptors from AGM COS7 cells (AGMPAR-1 and AGMPAR-2), rhesus macaque FRhK4 cells (rhPAR-1 and rhPAR-2), cynomolgus macaque primary splenocytes (cynPAR-1 and cynPAR-2), and baboon primary PBMC (baPAR-1 and baPAR-2). Based on these sequences, quantitative RT-PCR was set up to determine expression levels of the receptor and compare them to those of PERV-A susceptible human cell lines 293T, HT1080, HeLa, and primary PBMC.

**Figure 3:**
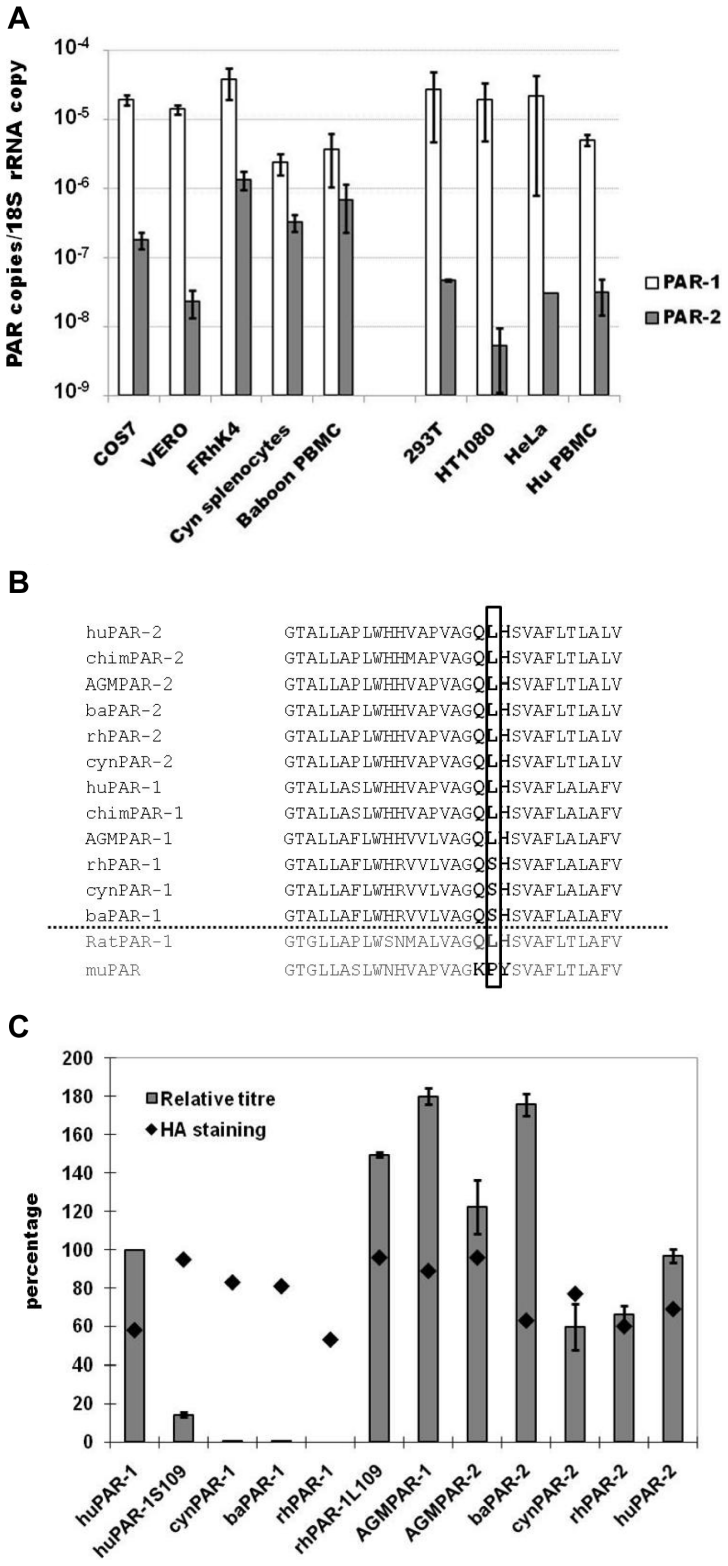
Endogenous expression, sequence and function of NHP PERV-A receptors. **A**) Total RNA was extracted from NHP and human cells and analysed by quantitative RT-PCR. Primers used for amplification of NHP PAR-1 (white bars, left) huPAR-1 (white bars, right) NHP PAR-2 (grey bars, left) and human PAR-2 (grey bars, right) are listed in Table S1. PAR copy number was calculated from standard curves and normalised per 18S rRNA copy. Histograms represent average of at least two independent experiments (± SEM). **B**) Human (huPAR-1 and huPAR-2), mouse (muPAR), rat (ratPAR), chimpanzee (chimPAR-1 and chimPAR-2), rhesus macaque (rhPAR-1 and rhPAR-2) PAR and baboon PAR-2 (baPAR-2) sequences were present in the NCBI database. African green monkey (AGMPAR-1 and AGMPAR-2), cynomolgus macaque (cynPAR-1 and cynPAR-2) and baboon (baPAR-1 and baPAR-2) PAR sequences were obtained by RT-PCR of RNA extracted from NHP cell lines and primary cells using specific primers (Table S1). Deduced amino acid sequences were aligned using ClustalW software. The three amino acids different between muPAR and huPARs are in bold and the amino acid 109 critical for PERV-A receptor function as previously described [42] is boxed. **C**) C-terminal HA-tagged PAR sequences were cloned into a MLV-based vector and introduced into quail QT6 cells by transduction of VSV-G pseudotyped retroviral particles. Percentage of HA-positive cells was measured by cell surface staining of the transduced cells and flow cytometry analysis (black diamond). 5×10^4^ PAR expressing QT6 cells were seeded and the day after infected with serial dilution of EGFP(PERV)-containing supernatant. After 72 hrs, EGFP expression was monitored and titers inferred. EGFP(PERV) titer on huPAR-1 expressing QT6 cells was 1.7±0.5×10^4^ Etu/mL and arbitrarily chosen as 100% infection. Histograms represent the average of three independent experiments (± SEM).

PAR-1 mRNA levels were found to be similar in NHP and human cells. Primary cells express about five times less PAR-1 mRNA than the cell lines (Figure 3A). The amount of PAR-2 mRNA was more variable. All human cells and AGM VERO had a low level of PAR-2, at least 2.5 orders of magnitude less than PAR-1. Instead, FRhK4 cells, primary baboon PBMC, and cynomolgus splenocytes expressed 10 times more PAR-2 mRNA than human cells and VERO cells (Figure 3A).

### Serine109 renders rhPAR-1, cynPAR-1, and baPAR-1 unable to mediate PERV-A entry

PAR amino acid sequences deduced both from our NHP PAR sequences and NCBI database sequences were aligned using the ClustalW programme. The extracellular domain 2 (ECL2) was located according to the huPAR-2 topology previously predicted [42]. The sequence of ECL2 was well conserved among different species with the exception of a serine instead of a leucine at amino acid (a.a.) 109 in rhPAR-1, cynPAR-1, and baPAR-1 (Figure 3B). This mutation was of particular interest because this position 109 has previously been shown to be critical for PERV-A infection and binding [42], [43].

The importance of a.a. 109 has been demonstrated by our finding that murine PAR containing proline at this position is inactive as a PERV receptor, and that leucine-to-proline mutations at a.a. 109 of huPAR-1 and -2 inactivate receptor activity. This suggests that the PERV receptor function of PAR-1 containing serine 109 may be impaired. To test the ability of the NHP receptors to support PERV-A entry, NHP PARs were HA-tagged at the C-terminus and subcloned into an MLV-based retroviral vector. PERV-A resistant quail QT6 cells were transduced using VSV-G pseudotyped MLV particles carrying PAR genes. More than 50% of the cells expressed the receptors as assessed by flow cytometry analysis after immunostaining with an anti-HA antibody (Figure 3C, diamond). PAR-expressing cells were infected with PERV-A14/220 carrying the EGFP gene (EGFP(PERV)), and the PERV infection titer determined by monitoring EGFP signal. The titers have been represented as percentage of that obtained on QT6/huPAR-1 cells (Figure 3C). All the PARs tested conferred permissivity to PERV-A entry in QT6 cells, with the exceptions of cynPAR-1, baPAR-1, and rhPAR-1. These receptors share the serine at a.a. 109, which is different from the other receptors (Figure 3B). To test whether the L109S change was responsible for the inability to support PERV-A entry, a huPAR-1 mutant carrying a serine instead of leucine in ECL2 (huPAR-1S109) and a rhPAR-1 with the opposite mutation (rhPAR-1L109), were generated. Once expressed in QT6 cells rhPAR-1L109 could efficiently mediated PERV-A entry. In contrast, the ability of huPAR-1S109 to function as receptor was reduced to 15% (Figure 3C). The a.a. substitution in position 109 appeared to have a negative effect on receptor function. These results suggest that PERV-A may enter rhesus monkey FRhK4 cells only through the poorly expressed PAR-2, providing a reason for the low susceptibility to PERV infection. This genetic trait should influence PERV infection to be inefficient in all baboon, rhesus, and cynomolgus macaque cells *in vivo* and *in vitro*.

### Tunicamycin treatment of AGM cells increases PERV-A infection

**Figure 4:**
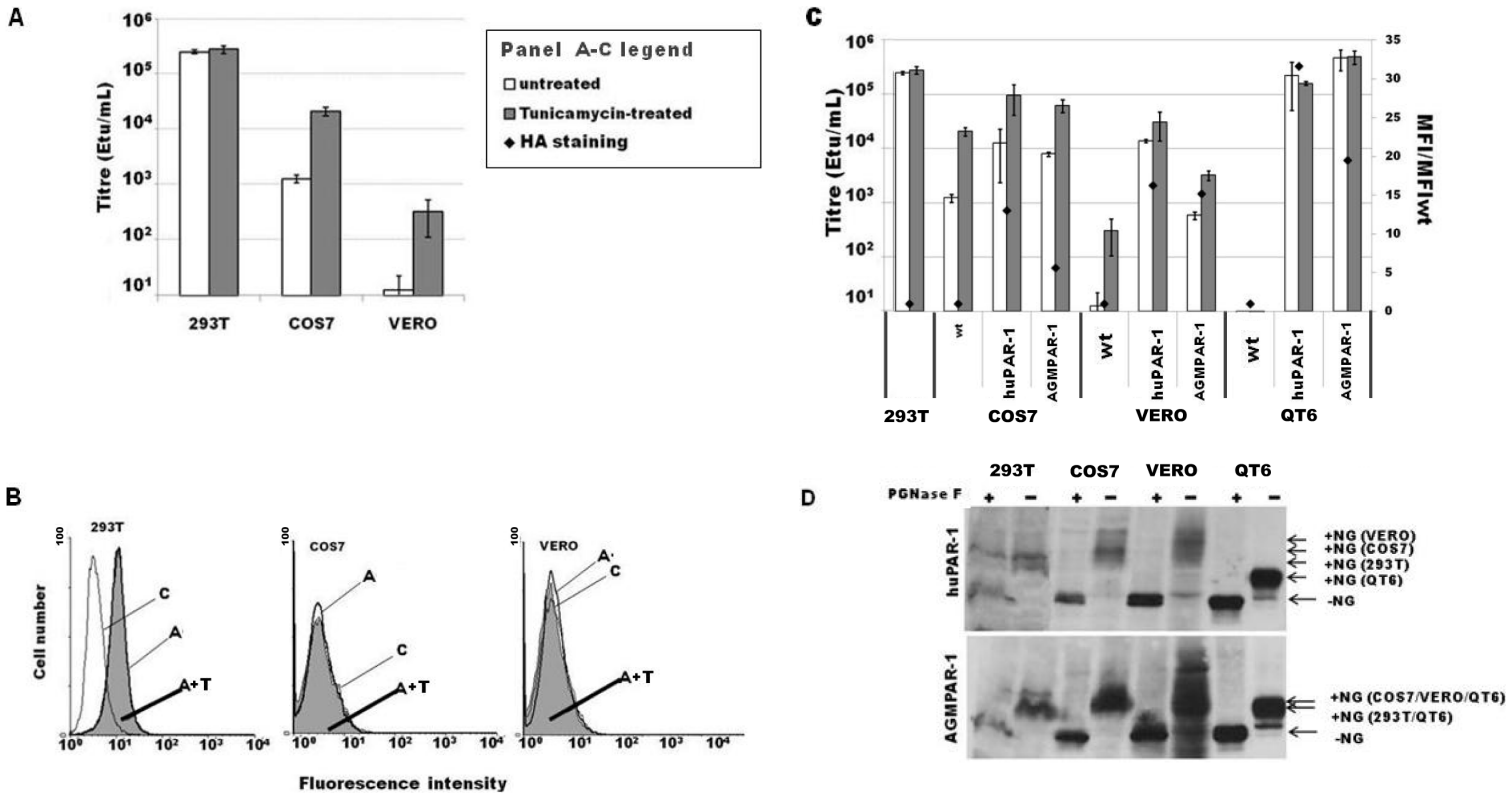
Effect of tunicamycin treatment on PERV infection and analysis of N-glycosylation of PERV-A receptors. Cells were treated for 16 hrs with tunicamycin at the final concentration of 100 ng/mL (293T cells), 200 ng/mL (COS7 and VERO cells) or 25 ng/mL (QT6 cells). **A**) Tunicamycin-treated (grey) or untreated (white) cells were infected with serial dilution of EGFP(PERV) and the titers calculated 72 hrs later by flow cytometry analysis. Histograms represent the average of four independent experiments (± SEM). **B**) Cells were incubated with 100 ng in 0.5 mL of soluble PERV-A360 Env or PERV-C360 Env and Env binding was detected using a FITC-conjugated anti-rabbit IgG antibody and flow cytometry analysis. A representative set of results of three independent experiments are shown for tunicamycin-treated cells with PERV-A360 Env (A+T), untreated cells with PERV-A360 Env (A), untreated cells with PERV-C360 Env (C) is shown. Almost identical histograms were obtained for PERV-C360 binding with and without tunicamycin treatment. **C**) Quail QT6, AGM COS7 and VERO cells were stably transduced with MLV-vectors carrying HA-tagged huPAR-1 or AGMPAR-1. More than 98% of the cells were positive for anti-HA antibody staining (data not shown). Mean fluorescence intensity (MFI) of anti-HA staining for each population was normalised to the MFI of the wild type cells (black diamond). Tunicamycin-treated (grey) or untreated (white) cells were infected with serial dilutions of EGFP(PERV) and the titers determined by EGFP expression monitored by flow cytometry analysis. Histograms represent the average of two independent experiments (± SEM). **D**) HA-tagged huPAR-1 (upper panel) and AGMPAR-1 (bottom panel) transduced cells were lysed in RIPA buffer and treated (+) or not (−) with PNGase F. Proteins were separated in a 10% SDS-PAGE and immunoblotted using an anti-HA antibody. The N-glycosylated (+NG) and the de-glycosylated (−NG) forms are indicated.

AGM COS7 and VERO cells express two functional receptors (Figure 4), and their mRNA was expressed at a similar level to highly permissive human cell lines (Figure 3B). Therefore, there may be other reasons why PERV-A poorly infects AGM cells. Removal of N-linked glycosylation by tunicamycin treatment of the target cells has been shown to rescue retroviral infectivity in certain cell lines [44], [45], [46]. To test whether N-linked glycosylation could play a role in the low susceptibility of AGM cells to PERV-A14/220 infection, 293T, COS7, and VERO cells were infected with serial dilutions of EGFP(PERV) after overnight treatment with tunicamycin. The viral titer on tunicamycin-treated AGM cells was more than 10-fold higher than untreated cells, whereas it had no effect on 293T cells (Figure 4A). These data suggested that removal of N-linked glycosylation in AGM cells could relieve a possible block to PERV-A infection. To better understand the role of tunicamycin in the improvement of the susceptibility to PERV, we analysed the effect this drug had on soluble PERV-A Env binding on AGM cells. PERV-A Env, but not PERV-C Env, successfully bound to 293T cells, with no difference after tunicamycin treatment. No binding was detected to either tunicamycin-treated or untreated AGM cells (Figure 4B). However, the lack of binding could be due to low assay sensitivity, as noted previously [42].

To understand whether the tunicamycin effect was receptor-specific or cell-specific, HA-tagged huPAR-1 and AGMPAR-1 were stably overexpressed on COS7 and VERO cells as well as on quail QT6 cells. More than 98% of the cells expressed the HA-tagged receptors, and its level on the cell surface was determined by the mean fluorescence intensity (MFI) after anti-HA immunostaining (Figure 4C, diamond). Whilst PERV infection of 293T and quail QT6 cells was not affected by tunicamycin treatment, the susceptibility of AGM cells to PERV-A14/220 infection was increased. This was regardless of which receptor, huPAR-1 or AGMPAR-1, was expressed (Figure 4C), suggesting that partial rescue of PERV infection by tunicamycin in AGM cells was cell-specific, and did not depend on the receptor expressed. Indeed, the HA-tagged receptors from the different cell lines without tunicamycin treatment showed different patterns by western blot indicating cell-dependent, differential, glycosylation (Figure 4D).

## Discussion

Our initial study on the PERV-A14/220 infection time course confirmed that it poorly infects and replicates in NHP cells compared to that in human 293T cells (Figure 1). This study also indicated that there are at least two steps at which PERV replication is blocked in NHP cells: cell entry and exit.

At the cell exit step, PERV-A14/220 appeared to be released much less effectively from NHP cells than from human 293T cells. Whilst processed PERV capsid (p27) produced by 293T cells were found mostly in the culture supernatant, the majority of p27 produced by NHP cells was associated with cells and not released to the supernatant (Figure 2A). This suggests that the matured virions are retained on the NHP cell surface, reminiscent of a tetherin-mediated inhibition of retroviral release [40], [47]. Consistent with the possibility that tetherin causes poor PERV release in NHP cells, the amount of tetherin mRNA in the NHP cell lines was 10–100 times higher than in the 293T cells (Figure 2B). However, the relevance of this finding to the *in vivo* context is unclear, as the control of tetherin expression, which is class type I interferon-inducible, is different *in vivo* from that in the cell lines used in this study. Though the *in vivo* tetherin expression in NHP has yet to be investigated, species difference compared to humans may be insignificant.

The block at the cell entry step is critical and, in the case of macaques and baboons, we identified a genetic defect in a PERV-A receptor gene, PAR-1. Firstly, expression of a functional human PERV-A receptor rendered NHP cell lines more amenable for viral entry and propagation (Figure 1), indicating that entry of the virus is a critical step in the low permissivity of NHP cells. Our study on cloned PAR-1 showed that rhesus macaque, cynomolgus monkey, and baboon PAR-1 genes encode serine at a.a. 109, and are unable to support PERV-A14/220 infection (Figure 3B and C). PERV poorly infects rhesus macaque cells probably via rhPAR-2, whose expression is lower than that of PAR-1 (Figure 3A). This PERV-A14/220 infection route is likely to be minor in humans, as huPAR-1 expression is more robust than that of huPAR-2 in most tissues *in vivo* [42]. These NHP species therefore lack the major infection route potentially used by PERV in humans. This fact must be taken into account in pig-to-NHP transplantation, where cynomolgus macaque and baboon are currently the most often used NHP species [48], and have been preferentially employed in PERV transmission studies *in vivo* [28], [29], [30], [31], [32]. The negative PERV transmission results in such experiments should be interpreted with caution.

Intriguingly, both AGMPAR-1 and AGMPAR-2 are able to support PERV-A infection (Figure 3C). When the same amount of huPAR-1 and AGMPAR-1 was expressed on the cell surface of PERV-A-resistant QT6 cells, the efficiency of PERV-A14/220 mediated-EGFP transduction was similar (Figure 4C), suggesting a comparable affinity of the receptors for the virus. However, AGM cells are poorly infected by PERV-A14/220. No evidence of an Fv1/TRIM5α-like restriction activity was found (data not shown), consistently with previous data in the literature [37], [49]. No inhibitors secreted from AGM cells were detected in the supernatant of these cells (data not shown). Tunicamycin treatment to inhibit N-glycosylation in the target cells could, however, rescue PERV-A infectivity (Figure 4A). This effect was cell-specific rather than receptor-specific (Figure 4C), and it correlated with cell differences in PAR glycosylation, i.e. a heavier glycosylation of the receptors in NHP cells than in 293T and QT6 cells (Figure 4D). A possible explanation for the tunicamycin-mediated increase in PERV-A infectivity is that heavy N-glycosylation of the receptor could prevent PERV binding, and that tunicamycin treatment could relieve this block. This was, however, not supported by our Env binding assay, since tunicamycin treatment did not increase Env binding to AGM cells (Figure 4B). Further investigation should be conducted to clarify the mechanism behind the poor permissivity of AGM cells to PERV-A. A limit of this study is the use of different cell lines between human and NHP. PERV-A infection conducted in parallel using the same primary human and NHP cells such as PBMC could provide more information on the suitability of AGM as animal model in xenotransplantation.

Here, infection of human-tropic, recombinant PERV-A/C in NHP cells has been examined, and several steps have been identified which are responsible for the lower efficiency of infection compared to that of human cells. PERV entry is inefficient in rhesus macaque cells because of the defect in PAR-1 PERV-A receptor function. The same is predicted for baboon and cynomolgus monkey cells, which have the same defective mutation. A genotypic analysis of the PAR sequences in the candidate species to be employed in pig-to-NHP transplantation will provide useful information on the likelihood that an animal may be as susceptible as humans to PERV infection. Indeed, AGM has no such defect in the PAR-1 gene, suggesting possible advantage for the use of this species.

## Materials and Methods

### Cell lines

Human embryonic kidney 293T cells [50] were maintained in Dulbecco's modified Eagle Medium (DMEM, Gibco) supplemented with 15% fetal bovine serum (FBS, BioSera). Quail QT6 cells (American Type Culture Collection (ATCC), CRL-1708), African green monkey COS7 (ATCC, CRL-1651) and VERO cells (ATTC, CCL-81), and rhesus macaque FRhK4 cells (ATCC, CRL-1688) were grown in DMEM supplemented with 10% FBS.

Primary baboon (*Papio anubis*) and cynomolgus macaque (*Macaca fascicularis*) cells were obtained at the Institut de Transplantation et de Recherche en Transplantation-Université de Nantes, France, according to the standard procedure of the EU FP6 Consortium Xenome project LSHB-CT-2006-037377 approved by the European Commission.

### Plasmids and virus production

Production of EGFP-carrying PERV-A [EGFP(PERV)] has been previously described [42]. Replication-competent PERVA14/220 was produced by transfection of 293T cells with 18 µL of FuGene-6 (Roche) and 3 µg of the plasmid pCRPERVA14/220 [38]. Viral particles carrying the receptor genes were produced by three plasmid transfection, as previously described [42]. The following expression plasmids, pcDNA3-huPAR1HA and pcDNA3-huPAR-2HA, and the retroviral vectors pCFCRhuPAR-2, pCFCRhuPAR-1HA, pCFCRhuPAR-2HA have been previously described [42]. To facilitate the production of HA-tagged NHP PARs, a ClaI restriction site was introduced, in frame, upstream of the HA-tag sequence in the pcDNA3-huPAR2HA (pcDNA3/huPAR-2ClaHA) by PCR-based mutagenesis using complementary primers C11–C12, in association with primers C3–C13 (Table S1) as previously described [42].

### Cloning of NHP PERV-A receptor and tetherin genes

Total cellular RNA was extracted using the RNeasy kit (Qiagen) and reverse transcribed as previously described [42]. NHPPAR and NHP tetherin sequences were then amplified using HotStart polymerase (Qiagen) and the following primer pairs (Table S1): C1–C2 (AGMPAR-1), C5–C2 (rhPAR-1, cynPAR-1, baPAR-1), C3–C4 (AGMPAR-2), C6–C7 (rhPAR-2, cynPAR-2, baPAR-2), C9–C10 (NHP tetherin). PCR products for NHP tetherins were cloned into a pGEM-T easy vector (Promega). C-terminal HA-tagged receptors were produced by introducing the PCR product into pcDNA3/huPAR-2ClaHA using EcoRI and ClaI restriction sites present in the forward and reverse primer, respectively. The mutant receptors huPAR-1S109 and rhPAR-1L109 were generated by PCR-mutagenesis using the following primer pairs (Table S1): C16–C17 and C18–C19 (huPAR1S109) or C14–C15 and C2–C5 (rhPAR-1L109). Finally, HA-tagged receptors were then subcloned in the MLV-based retroviral vector pCFCR using the restriction sites EcoRI and NotI.

### Transduction and infection assay

1×10^5^ cells were infected with 1 mL of the PERVA14/220-containing supernatant (MOI ∼20) in the presence of 8 µg/mL polybrene. Cells were kept in culture for 4 weeks. At different time points, 1×10^6^ PERV-infected cells were seeded in a 6-well plate and, the day after, serial dilutions of their supernatant were used to infect 3×10^4^ 293T cells. After 72 hrs, the titer was determined by *in situ* immunostaining of infected cells as previously described [39]. The receptor transduction and EGFP(PERV-A) infection were performed as follows: 5×10^4^ target cells were seeded in a 12-well plate and the day after, 500 µL of virus-containing supernatant and 8 µg/mL of polybrene were added. HA-tagged receptor or EGFP expression was verified 48 hrs post transduction/infection by flow cytometry analysis, as previously described [42].

### Soluble Envelope Binding Assay

Expression plasmid for soluble PERV-A360 and PERV-C360 Env fused to rabbit immunoglobulin γ-heavy chain (rIgG) were a kind gift from Dr C Wilson [51]. Soluble proteins were produced by transfection of 293T cells with pSKPERV-A360 or pSKPERV-C360 plasmid. Binding assay was performed as previously described [51].

### Immunoblotting

1×10^6^ PERV-infected cells were lysed as previously described [42]. Virus particles in the supernatant of virus-producing cells were concentrated by a centrifugation for 4 hrs at 16000G at 4°C. For the glycosylation assay, a quarter of the total cell lysate was digested with 1500U of N-glycosidase F enzyme (PGNase F, New England Biolabs) at 37°C for 2 hrs. The whole reaction of digested proteins, a quarter of the other cell lysates and of the concentrated supernatant, was used for immunoblotting as previously described [42], [47], using the monoclonal antibody HA.11 (Covance) or an anti-PERV capsid antibody [39].

### Quantitative RT-PCR

Quantitative RT-PCR was conducted as previously described [47]. The amount of RNA between each samples was normalized using the housekeeping gene 18S rRNA. The assay was performed in duplicate using the Eppendorf RealPlex 4 as previously described [47]. Used as a copy number standards are: pCR-PERVA14/220 (PERV *gag*), pGEM-rhesus macaque tetherin and pGEM AGM tetherin (NHP tetherin), human tetherin-expressing plasmid [40], pcDNA3-huPAR1HA, pcDNA3-huPAR-2HA, pcDNA3/HA expressing NHP PARs (described above), TOPO-18S rRNA gene [42].

### PERV-A receptors accession number

PERV-A receptor amino acid sequences were derived from the following nucleotide sequences. Already present in the NCBI database prior to this study were: human PAR-1 [AY070774] and PAR-2 [AY070775], chimpanzee PAR-1 [XM_001156784] and PAR-2 [XM_001164395], rhesus macaque PAR-1 [XM_001091189] and PAR-2 [XM_001099620], murine PAR [NM_029643.3] and rat PAR [NM_001109670]. The following nucleotide sequences were determined in this study and deposited in the GenBank database: AGMPAR-1[HM347351], AGMPAR-2 [HM347352], baboon PAR-1 [HM347353], baboon PAR-2 [HM347354], cynomolgus macaque PAR-1 [HM347355] and PAR-2 [HM347356].

## Supporting Information

Table S1 Primers used in this study.
(0.05 MB DOC)
