# Supplementary material for: Suboptimal Porcine Endogenous Retrovirus Infection in Non-Human Primate Cells: Implication for Preclinical Xenotransplantation
Source: PLoS One. 2010 Oct 6;5(10):e13203. doi: 10.1371/journal.pone.0013203 (PMC2950858; doi:10.1371/journal.pone.0013203)
Supplement: Table S1 — Primers used in this study. (0.05 MB DOC) [file pone.0013203.s001.doc]

**TABLE**

**Table S1. Primers used in this study**.

|  | **Cloning primers** | **Target** |
| --- | --- | --- |
| C1 | GTT CCA GAA TTC^a^ ACC ACC ATG G^b^CA GCA CC ACA CCC GGC | AGMPAR-1 |
| C2 | CAA GGA TCG AT^c^G GAG TCA CAG GGG TCT GC | AGMPAR-1, rhPAR-1, cynPAR-1, baPAR-1 |
| C3 | GAT TGA TGA ATT C^a^AC CAC CAT GG^b^C AGC ACC CAC G | AGMPAR-2 |
| C4 | AAC ATC GTA TGG GTA ATC GAT^c^ GGG GCC ACA GGG GTC | AGMPAR-2 |
| C5 | GTT CCA GAA TTC^a^ ACC ACC ATG G^b^CA GCA CCC ACA CCC AGC | rhPAR-1, cynPAR-1, baPAR-1 |
| C6 | GTC CAG AAT TC^a^A CCA CCA TGG^b^ CAG CAC CCA TG | rhPAR-2, cynPAR-2, baPAR-2 |
| C7 | CAA GGA TCG AT^c^G GGG CCA CAG G | rhPAR-2, cynPAR-2, baPAR-2 |
| C9 | ATG GCA CCT ATT TTG TAT GAC TAT | NHP tetherin |
| C10 | TCA CAG CAG CAG AGC | NHP tetherin |
|  | **Mutagenesis primers** |  |
| C11 | GAC CCC TGT GGC CCC ATC GAT^c^ TAC CCA TAC GAT GTT | pcDNA3/huPAR-2ClaHA |
| C12 | AAC ATC GTA TGG GTA ATC GAT^c^ GGG GCC ACA GGG GTC | pcDNA3/huPAR-2ClaHA |
| C13 | GTT CTT TCC GCC TCA GAA GC | pcDNA3/huPAR-2ClaHA |
| C14 | GCA GGA CAG TTG CAT TCC GTG | rhPAR-1L109 |
| C15 | CAC GGA ATG CAA CTG TCC TGC | rhPAR-1L109 |
| C16 | GCA GGA CAG TCG CAT TCT GTG G | huPAR-1S109 |
| C17 | CCA CAG AAT GCG ACT GTC CTG C | huPAR-1S109 |
| C18 | CGA CTC ACT ATA GGG AGA CCC | huPAR-1S109 |
| C19 | TTA GGA AAG GAC AGT GGG AGT | huPAR-1S109 |
|  | **Real-time PCR primers** |  |
| Q1 | AGC CTA CTT GGG ATG ATT GTC AA | PERV *gag* |
| Q2 | GGC CCC AGG AAC ATT TTT TC |  |
| Q3 | ACC TGC AAC CAC ACT GTG ATG | Human tetherin |
| Q4 | CAA GCT CCT CCA CTT TCT TTT GTC |  |
| Q5 | ACC TGC AAC CAG ACT GTG ATG | NHP tetherin |
| Q6 | CAA GCT CCT CCA CTT TCT TTC GTC |  |
| Q7 | CTG CCT TCC AGG GTC TTC TG | huPAR-1 |
| Q8 | TGA GGA CTC TTC CAC CTC TTC CT |  |
| Q9 | TCA GGG CCT GAA CTT CAA CTG | huPAR-2 |
| Q10 | GCA ATG GCA AAG CCT CTT CT |  |
| Q11 | GTA CCC ACA GGG GGC TTA GGA TC | NHPPAR-1 |
| Q12 | CTT GGT GGC TCT TGC AAT GGT G |  |
| Q13 | GCA GGG CCT GAA CTT CCA TTG | NHPPAR-2 |
| Q14 | AAG GCA CCCA TGG GCT GAG AAC |  |
| Q15 | TCG AGG CCC TGT AAT TGG AA | 18S rRNA |
| Q16 | CTT GCC CTC CAA TGG ATC CT |  |

^a^ EcoRI; ^b^ Kozak sequence; ^c^ ClaI.
